# Incidence of Liver Damage of Uncertain Origin in HIV Patients Not Co-Infected with HCV/HBV

**DOI:** 10.1371/journal.pone.0068953

**Published:** 2013-07-18

**Authors:** Antonio Rivero-Juárez, Angela Camacho, Nicolás Merchante, Inés Pérez-Camacho, Juan Macias, Carmen Ortiz-Garcia, Celia Cifuentes, Julián Torre-Cisneros, José Peña, Juan A. Pineda, Antonio Rivero

**Affiliations:** 1 Infectious Diseases Unit, Instituto Maimonides de Investigación Biomédica de Córdoba (IMIBIC), Hospital Universitario Reina Sofia, Cordoba, Spain; 2 Infectious Diseases Unit, Instituto de Investigación Sanitaria de Sevilla (IBIS), Hospital Universitario de Valme, Seville, Spain; 3 Internal Medicine Department, Hospital de Poniente, Almeria, Spain; 4 Clinical Analysis Unit, Instituto Maimonides de Investigación Biomédica de Córdoba (IMIBIC), Hospital Universitario Reina Sofia, Cordoba, Spain; 5 Immunology Unit, Instituto Maimonides de Investigación Biomédica de Córdoba (IMIBIC), Hospital Universitario Reina Sofia, Cordoba, Spain; University of Modena & Reggio Emilia, Italy

## Abstract

**Background and Aims:**

Several studies have reported that a significant number of HIV patients not co-infected with HCV/HBV develop liver damage of uncertain origin (LDUO). The objective of our study was to evaluate the incidence of and risk factors for the development of LDUO in HIV infected patients not co-infected with HCV/HBV.

**Methods:**

Prospective longitudinal study that included HIV-infected patients free of previous liver damage and viral hepatitis B or C co-infections. Patients were followed up at 6-monthly intervals. Liver stiffness was measured at each visit. Abnormal liver stiffness (ALS) was defined as a liver stiffness value greater than 7.2 kPa at two consecutive measurements. For patients who developed ALS, a protocol was followed to diagnose the cause of liver damage. Those patients who could not be diagnosed with any specific cause of liver disease were diagnosed as LDUO and liver biopsy was proposed.

**Results:**

210 patients matched the inclusion criteria and were included. 198 patients completed the study. After a median (Q1–Q3) follow-up of 18 (IQR 12–26) months, 21 patients (10.6%) developed ALS. Of these, fifteen patients were diagnosed as LDUO. The incidence of LDUO was 7.64 cases/100 patient-years. Histological studies were performed on ten (66.6%) patients and all showed liver steatosis. A higher HOMA-IR value and body mass index were independently associated with the development of LDUO.

**Conclusion:**

We found a high incidence of LDUO in HIV-infected patients associated with metabolic risk factors. The leading cause of LDUO in our study was non-alcoholic fatty liver disease.

## Introduction

Liver disease is a leading cause of death among human immunodeficiency virus (HIV)-infected patients receiving highly active antiretroviral treatment (HAART) [1], [2]. Among the main causes of liver disease, co-infection with the hepatitis C (HCV) or hepatitis B (HBV) virus is the most frequent and has the worst prognosis [3]. However, HIV-infected patients without viral hepatitis co-infections have been identified who develop severe liver damage that progresses to fibrosis, cirrhosis and even terminal liver disease [4]–[7]. In such HIV-infected patients, transient liver elastography (TLE) improves the chances of screening for liver damage [8]. Studies carried out on HIV-infected patients using TLE have reported that liver damage is widespread in this population [8], [9]. Known related causes are alcohol abuse and prolonged exposure to various antiretroviral drugs, especially didanosine (ddI) [8]–[10]. Partly because of this, the use of ddI and stavudine (d4T) is not currently recommended [11]. The incidence of and risk factors for liver damage of uncertain origin (LDUO) among HIV-infected patients have not been identified in the context of non-use of ddI or alcohol. We designed therefore a prospective study with a cohort of HIV-infected patients not co-infected with HCV/HBV to evaluate the incidence of LDUO, assess the factors associated with it and determine the aetiology of these cases.

## Materials and Methods

### Ethical Aspects

The study was designed and performed according to the Helsinki Declaration and approved by the ethical committee of the Reina Sofia University Hospital, Cordoba, Spain. All patients provided written informed consent before participating in the study and before liver biopsy was performed.

### Study Design

Included in this longitudinal prospective study were Caucasian HIV type-1-infected patients attending three reference hospitals in southern Spain who were followed up between January 2009 and December 2011.

### Selection of Patients

At the time of inclusion in the study, patients completed a questionnaire, which included self-reported daily alcohol consumption, and underwent a clinical examination and routine haematological, biochemical, immunological and virological assessments to rule out potential causes of liver disease. The key criteria for inclusion were: older than 18 years of age; HIV infection confirmed by the Western Blot test; no current clinical conditions associated with HIV; no HBV and/or HCV co-infection, defined as the absence of specific anti-HBV (HBsAg) and HCV antibodies, and plasma unquantificable HCV-RNA and HBV-DNA by PCR; and an LS value of less than 7.2 kPa at the baseline visit. The key criteria for exclusion were: evidence of liver disease; previous or current use of ddI or d4T; use of other potentially hepatotoxic drugs and alcohol consumption higher than 20 gr/day.

### Follow-up

All patients included in the study were followed up every 6 months. At each visit, the following data were collected: Analytical variables (unit): ALT (IU/L), AST (IU/L), GGT (IU/L), total fasting cholesterol, fasting LDL cholesterol, fasting triglyceride levels (mg/dL), platelet count (10^6^/µL), glucose (mg/dL), and insulin (mg/dL); Variables relating to HIV infection (unit): HIV viral load (copies/mL), CD4+ count (cells/mL), AIDS-defining condition, time since HIV diagnosis and HAART regimen; Demographics (unit): body weight (Kg), height (cm), alcohol consumption (gr/day) and concomitant medication. HIV viral load was measured by PCR (Cobas TaqMan, Roche Diagnostic Systems Inc., Pleasanton, CA, USA), and the detection limit set at 20 IU/mL. Samples were tested in the clinical analysis units of the three participating hospitals. After an overnight fast of at least 12 h, venous blood was drawn to measure glucose in the fresh samples. Serum was separated from part of every sample and immediately frozen at −20°C. The serum was thawed at one clinical analysis unit (Hospital Reina Sofia de Cordoba) to determine the serum level of insulin, using standard laboratory techniques,.

At every visit, TLE was used to obtain liver measurements of all patients included in the study. The measurements were taken by a single experienced operator at each participating hospital, using an M-probe, following a routine described elsewhere, [12], [13]. At least 10 valid LS measurements were obtained from each patient before the examination ended. Only those examinations with an interquartile range (IQR) of <30% of the median value and a successful acquisition rate of >60% were considered for analysis [13].

Body mass index (BMI) and homeostasis model assessment-insulin resistance (HOMA-IR) were calculated using the specific variable data collected. A HOMA-IR score of more than 2.6 was considered abnormal [14].

### Evaluation of Liver Disease

ALS was defined as a liver stiffness value of more than 7.2 kPa, confirmed at two consecutive visits [8]. For those patients who developed ALS, a protocol was followed to diagnose the cause of liver damage: PCR was used to screen for HCV-RNA, HBV-DNA (Roche Diagnostic Systems Inc., Pleasanton, CA, USA) and HEV-RNA (Shanghai ZJ Bio-Tech Co., Ltd, China) to rule out occult viral hepatitis infection; ferritin, transferrin saturation, anti-liver autoantibody, anti-nuclear antibody, alpha-1-antitrypsin, copper and ceruloplasmin determinations were taken to screen for co-infection with hepatotropic viruses, haemochromatosis, autoimmune hepatitis, alpha-1-antitrypsin deficiency, or Wilson’s disease. Additionally, an abdominal ultrasound examination was carried out.

Those patients who could not be diagnosed with any specific cause of liver disease were diagnosed as LDUO and liver biopsy was proposed. Samples were analyzed by the same experienced pathologists at each centre who carefully searched for any recognizable cause of liver damage. The Knodell histology activity index, as modified by Scheuer, was used to assign a liver fibrosis score [15]. Nodular regenerative hyperplasia was defined as nodular architecture without extensive fibrosis or as the association of thickened and atrophic liver cell plates [16]. Liver steatosis was graded using Brunt et *al*’s criteria, and classified as follows: 0, steatosis absent; 1, less than 33%, or mild steatosis; 2, 33–66%, or moderate steatosis; 3, more than 66%, or severe steatosis [17].

### Statistical Analysis

Continuous variables were expressed as means (SD) or median and quartiles (Q1–Q3); the Student’s *t-*test, Welch test or Mann-Whitney *U*-test were used to compare two independent variables, and the Kruskal-Wallis, one-way ANOVA or Welch tests to compare more than two independent variables. The most appropriate test was chosen on the basis of a normal distribution (using the Shapiro-Wilk test) or equality of variances (using the Levene test). Categorical variables were expressed as number of cases (percentage). Frequencies were compared using the χ^2^ test or Fisher’s exact test. Significance was set at a *p* value of less than 0.05. A bivariate analysis was carried out to discover which variables were associated with ALS. Antiretroviral treatment drugs were analyzed according to their use and length of exposure. Logistic regression analysis was performed which included variables associated with the outcome variable in the bivariate analysis with *p* values of less than 0.2. The Hosmer-Lemeshow test was used to test for goodness of fit for the logistic regression model. The descriptive multivariate analyses followed Harrell FE Jr *et al*’s recommendations [18]. Analyses were carried out using the SPSS statistical software package, version 18.0 (IBM Corporation, Somers, NY, USA).

## Results

### Study Patients

One thousand two hundred and ninety-nine patients infected by HIV were tested at the three participating hospitals. Two hundred and ten patients matched the inclusion criteria and were enrolled in the study. One hundred and ninety-eight (90.6%) completed at least one follow-up visit. After a median (Q1–Q3) follow-up of 18 (IQR: 12–26) months, 21 patients (10.9%) presented ALS when assessed. After supplementary testing, a definitive diagnosis was reached for six (25.5%) of these patients (3 liver hemangiomas, 2 liver abscesses and 1 active hepatitis E virus infection). Consequently, the 15 remaining patients were diagnosed as LDUO. The median LS value in these patients was 9 kPa (IQR: 8.3–13.5 kPa).

### Incidence of LDUO and Associated Factors

The incidence of LDUO in our population was 7.64 cases/100 patient-years. The most relevant demographic, virological, and clinical characteristics are shown in Table 1. The LDUO group contained a higher proportion of patients with a baseline CD4+ count of less than 200 cells/mL (p = 0.049), higher BMI scores (p = 0.01), higher triglyceride levels (p = 0.03) and a higher percentage had HOMA-IR values of more than 2.6 (p<0.001) (Table 1). In our study, use of HAART drugs and length of exposure to drugs were not associated with ALS (Table 2).

**Table 1:** Baseline characteristics of patients with normal and abnormal liver stiffness values.

Characteristics	Non-LDUO(N = 177)	LDUO(N = 15)	P
Sex, no. (%)			
Male	117 (66.1)	10 (66.6)	0.96
Female	60 (33.9)	5 (33.4)	
Age (years). Mean (SD)	44.3 (9.7)	45.2 (7.4)	0.67
Undetectable HIV viral load, no. (%)*	135 (76.3)	11 (73.3)	0.86
AIDS defining criteria, no. (%)†			
No	130 (73.4)	12 (80)	0.60
Yes	47 (26.6)	3 (20)	
Nadir CD4 count (cells/mL). Mean (SD)	225.12 (176)	235 (160)	0.51
Baseline CD4 count (cells/mL). Mean (SD)	575 (291)	547·8 (360)	0.77
Baseline CD4 count lower than 200 cells/mL, no. (%)			0.049
No	166 (93.7)	12 (80)	
Yes	11 (6.3)	3 (20)	
Risk factor for HIV transmission, no. (%)			
IDU	1 (0.5)	0	0.94
Sexual	148 (83.6)	13 (86.6)	
Others‡	8 (4.6)	1 (6.7)	
Unknown	20 (11.3)	1 (6.7)	
Estimated time since HIV infection (years). Mean (SD)	8.89 (5.3)	8.35 (6)	0.74
BMI (m/Kg^2^). Mean (SD)	24.7 (4.1)	29.3 (5.6)	0.01
Fasting plasma cholesterol (mg/dL). Mean (SD)	202.4 (40.6)	206.4 (57.2)	0.7
LDLc (mg/dL). Mean (SD)	124.8 (36.04)	122.3 (45.8)	0.78
Triglycerides (mg/dL). (SD)	177.08 (131.6)	230.9 (90.3)	0.03
HOMA-IR. (SD)§	2.07 (1.39)	2.6 (1.04)	0.06
HOMA-IR higher than 2.6, no. (%)§			
Yes	41 (26.7)	11 (73.3)	<0.001
No	112 (73.3)	4 (26.7)	

*Abbreviations:* liver damage of uncertain origin (LDUO), human immunodeficiency virus (HIV), standard deviation (SD), acquired immunodeficiency syndrome (AIDS), injecting drug user (IDU), body mass index (BMI), low-density cholesterol (LDLc), homeostasis model assessment-insulin resistance (HOMA-IR).

* HIV viral load was measured by PCR (Cobas TaqMan, Roche Diagnostic Systems Inc., Pleasanton, CA, USA), detection limit set at 20 IU/mL.

† Classified on the basis of Center for Disease Control and Prevention (CDC) recommendations (Revised surveillance case definitions for HIV infection among adults, adolescents, and children aged <18 years and for HIV infection and AIDS among children aged 18 months to <13 years- United States, 2008. MMWR 2008; 57 (No RR-10): 1–14).

‡ Other causes of HIV transmission include: vertical transmission, blood transfusion, transplant and serovascular accident.

§ Data available for 168 patients.

**Table 2:** Association between length of exposure to antiretroviral drugs ever used and LDUO.

			Development of LDUO during the study					
			No (n = 177)	Yes (n = 15)	P	No (n = 177)	Yes (n = 15)	P
			Drug Used, no. (%)			Time (years), mean (SD)		
**Nucleoside Reverse Transcriptase Inhibitor**								
ABC		Yes	45 (25.4)	5 (33.3)	0.50	4.1 (2.6)	5.24 (2.6)	0.40
		No	132 (74.6)	10 (66.7)				
3TC		Yes	73 (41.2)	8 (53.3)	0.36	7.73 (4.01)	5.75 (3.64)	0.15
		No	104 (58.8)	7 (46.7)				
FTC		Yes	90 (50.8)	9 (60)	0.49	2.74 (1.48)	2.26 (1.13)	0.25
		No	87 (49.2)	6 (40)				
TDF		Yes	100 (56.5)	6 (40)	0.79	3.69 (2.2)	2.99 (1.91)	0.24
		No	77 (43.5)	9 (60)				
**Non-Nucleoside Reverse Transcriptase Inhibitors**								
EFV	Yes		59 (33.3)	3 (20)	0.28	5.68 (3.22)	2.93 (2.4)	0.1
	No		118 (66·7)	12 (80)				
ETV	Yes		7 (3.9)	0	0.43	1.88 (1.07)		
	No		170 (96.1)	15 (100)				
NVP	Yes		18 (11.3)	1 (6.6)	0.66	5.42 (3.9)	3 (0.122)	0.24
	No		159 (88.7)	14 (93.4)				
**Protease Inhibitors** *								
ATV	Yes		30 (16.9)	4 (26.6)	0.34	3.35 (2.03)	2.87 (0.405)	0.64
	No		147 (83.1)	11 (73.4)				
DRV	Yes		19 (10.7)	2 (13.3)	0.75	3.44 (3.65)	3.2 (2.89)	0.91
	No		158 (89.3)	13 (86.7)				
SQV	Yes		15 (8.5)	3 (20)	0.14	4.83 (3.4)	4.55 (1.82)	0.89
	No		162 (91.5)	12 (80)				
LPV	Yes		46 (25.9)	6 (40)	0.24	4.22 (2.67)	3.12 (1.38)	0.16
	No		131 (74.1)	9 (60)				
**CCR5 co-receptor antagonist**								
MVC	Yes		5 (2.8)	0	0.51	1.56 (1.16)		
	No		172 (97.2)	15 (100)				
**Integrase Inhibitor**								
RAL	Yes		20 (11.3)	2 (13.3)	0.81	1.78 (0.8)	1.82 (0.13)	0.84
	No		157 (88.7)	13 (86.7)				

*Abbreviations*: liver damage of unknown origin (LDUO), standard deviation (SD), Abacavir (ABC), lamivudine (3TC), emtricitabine (FTC), tenofovir (TDF), efavirenz (EFV), etravirine (ETV), nevirapine (NVP), atazanavir (ATV), darunavir (DRV) saquinavir (SQV), lopinavir (LPV), maraviroc (MVC), raltegravir (RAL).

* All protease inhibitors were boosted with ritonavir (100 mg).

In the logistic multivariate analysis for LDUO, independent risk factors were identified as BMI and a HOMA-IR score of more than 2.6 (Table 3).

**Table 3:** Multivariate logistic regression model for LDUO.

Characteristic	OR	CI 95%	P
Detectable HIV viral load	2.86	0.78–9.36	0.23
BMI	1.39*	1.06–1.82	0.018
Triglycerides	1.002	0.99–1.01	0.66
HOMA-IR higher than 2.6	4.41	1.14–16.99	0.03
3TC use	0.86	0.61–1.21	0.39
EFV length of exposure	2.38	0.24–22.08	0.46
LPV length of exposure	1.86	0.18–18.53	0.59
CD4 count less than 200 cells/mL	5.57	0.55–55.9	0.14

Hosmer-Lemeshow test: 0.530. R Nagelkerke: 0.240.

*Abbreviations:* liver damage of uncertain origin (LDUO), Odds ratio (OR), confidence interval (CI), human immunodeficiency virus (HIV), body mass index (BMI), Homeostasis model assessment-insulin resistance (HOMA-IR), lamivudine (3TC), efavirenz (EFV), lopinavir (LPV).

* The BMI OR shows the risk of developing LDUO per unit increase in BMI.

### Histological Findings

Of those patients who developed LDUO, ten (66.6%) agreed to undergo liver biopsy. The histological finding was liver steatosis in all of these patients: seven (70%) with grade 2 and three (30%) with grade 3 steatosis. Two (20%) of the patients had inflammatory activity. Liver fibrosis findings were: F2, three (30%) patients; F1, six (60%) patients; F0, one (10%) patient. All patients were diagnosed with non-alcoholic fatty liver disease (NAFLD).

## Discussion

Several studies have mentioned that a significant number of HIV patients not co-infected with HCV/HBV present LDUO, although so far no studies have evaluated the incidence of this (8, 9). As far as we are aware, ours is the first study to describe the incidence of LDUO in HIV-infected patients not co-infected with HCV/HBV and to identify NAFLD as the main cause of LDUO in this population.

The incidence of LDUO in our cohort was 7.64 cases/100 patient-years. Our findings showed that the main cause of LDUO in HIV-infected patients was associated with metabolic disorder. BMI (per unit increase in BMI) and elevated HOMA-IR values (higher than 2.6) were identified as the main risk factors for LDUO in HIV-infected patients. Liver biopsies were performed on ten (66.6%) of 15 LDUO patients and every histological examination showed liver steatosis. NAFLD was diagnosed as the main cause of LDUO in our study.

NAFLD is a common liver disease among the general population and has been increasing in developed countries in recent decades [19]. In histological terms, NAFLD is characterized by the excessive accumulation of lipids in hepatocytes, with or without inflammatory cell infiltration, necrosis or fibrosis. Studies reporting the incidence of NAFLD in the general population are scarce and the results deriving from them variable [20]–[22]. Two studies performed with Japanese populations reported incidence rates of 31 and 86 cases, respectively, of non-histologically confirmed NAFLD per 1,000 person-years [20], [21]; however, in another one, carried out by Wallis *et al*, in which a Caucasian population was used and required liver biopsy to confirm NAFLD, the incidence was lower (29 cases per 100,000 person-years) [22]. The incidence of and risk factors for NAFLD among HIV-infected patients have never been prospectively determined. Our study suggests that the development of NAFLD could be an emerging cause among HIV-infected patients.

HIV is present in the liver and can promote fibrosis by exerting indirect effects on the hepatocytes and/or directly triggering hepatocyte apoptosis [23]. However, the direct role of HIV on hepatic steatosis has not been clearly described. Several studies, conducted in both the pre-HAART and the HAART eras, have suggested that HIV infection plays an important role in the accumulation of fat in the liver and as a result plays a part in exerting steatosis activity [24]–[27]. Recently, a set of practice guidelines developed for NAFLD by the American Association for the Study of Liver Diseases (AASLD) has recommended screening for NAFLD in high-risk groups [28]. Our results lead us to pose the question of whether HIV-infected patients could be considered a risk group for NALFD and whether screening for it should be recommended, even among those without HCV/HBV co-infection.

Previous studies have highlighted the use of ddI as a risk factor for LDUO [4], [8]. The use of this drug, however, is currently restricted and ddI has been replaced by drugs with fewer adverse events and toxicity. In this new scenario, re-evaluating the risk factors associated with ALS is a key aspect. In our study, those ART drugs still in current use were not associated risk factors for the development of LDUO. On the other hand, BMI and HOMA-IR, which our study identified as risk factors for LDUO, have been highlighted as causes of NAFLD [29]. The actual diagnosis of NAFLD is difficult because screening tests are limited [28]. Our study suggests that, for HIV patients not co-infected with HCV/HBV, TLE could improve the chances of screening for liver steatosis.

Our study has a few limitations. Firstly, the threshold used to define LS as abnormal (a value of 7.2 kPa) was selected on the basis of previous information about the diagnostic yield of significant fibrosis using TE in HIV/HCV-co-infected patients [12]; as yet, however, no studies have established or validated specific cut-offs for ‘normal values’ of LS. Secondly, false positive TE results could have played a role in our results, although we can reasonably exclude this from our study because liver biopsy, when available, confirmed the presence of some degree of liver damage in every instance. Thirdly, liver histology was not available in all cases, since one-third of the patients refused to undergo liver biopsy. This is not surprising since liver biopsy is invasive and not always well tolerated by the patient. Fourthly, alcohol consumption was self-reported by the patient and this value may have been underestimated. Nevertheless, there is no objective method for determining the actual value of alcohol consumption. Fifthly, some HAART drugs are barely represented in our study, so that a better-powered cohort would be necessary to find significant associations. Lastly, in our study, one of the groups derived as a result of classifying on the basis of the presence or absence of LDUO as a dependent variable, contained only 15 patients. Because of its lack of power, therefore, our logistic regression model may have failed to identify variables which could in fact be associated with LDUO. However, our logistic model did lead us to identify the variables most strongly associated with LDUO (BMI and HOMA-IR).

In conclusion, our study described a high incidence of LDUO in HIV-infected patients with no previous liver damage. Secondly, metabolic risk factors were identified as risk factors for LDUO. Thirdly, the leading cause of LDUO in our study was NAFLD, and fourthly, current first-line ART drugs were not associated with the development of LDUO. These results may have important implications in caring for patients infected with HIV in everyday clinical practice.
